# Pupil dilation reflects English /l//r/ discrimination ability for Japanese learners of English: a pilot study

**DOI:** 10.1038/s41598-020-65020-1

**Published:** 2020-05-15

**Authors:** Yuya Kinzuka, Tetsuto Minami, Shigeki Nakauchi

**Affiliations:** 1 Department of Computer Science and Engineering, Toyohashi University of Technology, 1-1 Hibarigaoka Tempaku, Toyohashi, Aichi Japan; 2 Electronics-Inspired Interdisciplinary Research Institute, Toyohashi University of Technology, 1-1 Hibarigaoka Tempaku, Toyohashi, Aichi Japan

**Keywords:** Neurophysiology, Human behaviour

## Abstract

The importance of the English language has been increasing as various fields have become more globalized. When Japanese people try to acquire foreign language such as English, learners find it difficult to perceive speech-sounds such as the phonemes /l/ and /r/ that are absent in their native language (e.g., “light”/lάit/ and “right”/rάit/). Recent studies report that a unique sound that deviates from a repetitive background sound induces pupillary dilation response (PDR) regardless of whether attention is directed to the sound or not. In this study, we investigated whether deviation in higher-order processing such as language processing induces PDR, and the possibility of determining implicit subjective English proficiency. A behavioural auditory distinguishing ability test was performed prior to the main experiment to quantitatively evaluate participants’ ability to distinguish English words. Then, by conducting an oddball paradigm-employing stimulus including the phonemes /l/ and /r/ with simultaneous pupil diameter recording, a significant dilation was evoked by /l/-/r/ speech sounds presented as deviant stimuli. Moreover, a strong correlation between the PDR amplitude and participants’ ability to distinguish English words was found; that is, individuals with higher ability to distinguish such words displayed a prominent PDR. Also, the PDR difference between the two groups classified by discrimination ability suggests that PDR might be sensitive to higher-order characteristics involved in language processing, which is independent from the aspects of physical sound and cognitive load.

## Introduction

To deal with intensifying competition in the globalized modern information society, English is regarded as a highly practical language. As a result, the number of populations learning English as a second language is increasing remarkably^1^. Consequently, the improvement of English proficiency has become an important issue even in today’s Japan as the demand for English is rapidly increasing in various fields. Nevertheless, despite many years of study, many Japanese fare poorly in English proficiency.

One of the reasons acquiring English as second language is so difficult for Japanese is the difficulty in distinction of sounds absent in Japanese (e.g., /l/ and /r/, /s/ and th /θ/). Among them, the comprehension of words including the phonemes /l/ and /r/ is considered to be especially difficult to distinguish (e.g., glass and grass)^2,3^.

These learning problems due to discrimination difficulties between phonemes that are non-existent in the first language are not unique to Japanese speakers learning English, as, for example, they also occur among English speakers learning Mandarin^4^. In these cases, foreign sounds make new language acquisition difficult for adults. However, the ability to distinguish speech-sounds and phonemes is essential for speech perception and so is necessary for the improvement of English proficiency^4^.

To date, when Japanese learners practicing English listening skills, in most cases the learner attempts to discriminate between words including phonemes /l/ and /r/, and the learner is then informed whether their answers are correct or not^5^. However, this method requires a long time for the learner to acquire the ability to distinguish the foreign sounds.

Recent studies presented the potential that speech-sounds deviation by non-existent phonemes /l/ and /r/ can be extracted from biological signals. These studies have focused on subconscious perceptual learning incepted by biological signals, for instance, by showing the state of brain activity to the learner using neurofeedback technology combined with fMRI and EEG measurement technologies^5,6^. Shibata *et al*. used a visual stimulus, whose size corresponded to the real-time amplitude of the mismatch negativity (MMN): an auditory event-related potential that serves as an index of sound-discrimination accuracy. The participants were instructed to make the visual stimulus as large as possible while passively listening to English speech sounds. After few days of training, it is reported that the neurofeedback training helped the participants to achieve significant improvement in English speech differentiation ability^5^. As most of our human brain activity is beyond our conscious awareness, the possibility of acquiring new knowledge, such as foreign languages, without the awareness of “learning” may bring about a drastic change to the concept of learning itself.

Sensed subjective deviation or unnaturalness including auditory difference is reported to be reflected not only in the P300 ERP component and MMN, but also in the pupillary dilation reflex (PDR)^7,8^. Liao *et al*. reported significant pupillary dilation after a 2,000 Hz pure tone and white-noise were presented as deviant stimuli in an oddball paradigm against a 1,000 Hz pure tone used as repeated high-frequent stimuli^8^. Another study has shown that the pupil diameter seems to be sensitive to the novelty of a sound such as pink-noise, a ringtone, or the cry of a baby, and this response was reported to be induced also in infants^9^. These previous studies demonstrate that auditory deviation and sound saliency can be extracted as an objective index from pupillary response as well as from MMN. In other words, if English speech sounds that include the phonemes /l/ and /r/ are acoustically distinguishable, PDR may be induced by subjective deviation and thus could be observed by pupillometry. Moreover, if auditory deviation of English pronunciation appears in the PDR, it might be possible to establish a method to estimate subjective auditory distinguishing ability of words including phonemes /l/ and /r/ from non-contact pupil measurement, which has a much lower computational cost than fMRI or EEG.

The purpose of this study is to establish a reliable method to estimate subjective characteristics regarding English listening ability based on pupillometry and to reveal the cognitive mechanisms including language recognition and internal language processing. In this paper, we discuss results obtained from pupillary response measurements while auditory presenting English words including /l/ and /r/ to participants, and a method to assess participants’ subjective English listening ability to auditory distinguish words by pupillary responses.

## Materials and methods

### Participants

All experimental procedures were in accordance with the ethical principles outlined in the Declaration of Helsinki and approved by the Committee for Human Research at the Toyohashi University of Technology, and the experiment was strictly conducted in accordance with the approved guidelines of the committee. Informed written consent was obtained from participants after procedural details had been explained. Twenty-one Japanese monolingual speakers of Japanese (17 men, 4 women; age range: 21–30 years (M = 22.9; SD = 2.17)) took part in the experiment. They began studying English in junior-high school at about 12 years of age. Most of their exposure to English had taken place limited in classroom environments. Two monolingual participants had reported a history of living outside Japan for more than a year, although none reported fluency in English. One participant’s pupillary response data were excluded from pupil analyses due to eye blinks on more than 80% of trials which could not be interpolated in the pre-processing phase. All participants had normal or corrected-to-normal vision and no participants reported a history of hearing disorders.

### Stimuli

Stimuli used in the oddball task paradigm consisted of one pair, two utterances: speech synthesized English words “light” /lάit/ and “right” /rάit, ɹait/, spoken by a female voice, which were generated by the Google Text To Speech (iSpeech API) speech synthesis engine.

In order to equalize the intensity of the stimuli, Adobe Audition CC 2018 was used to adjust the average amplitude value in both of the stimuli. All generated stimuli were uniformed in American English, which is considered familiar to Japanese people. Stimuli were presented via loudspeakers located to the left and right of the screen, with an acoustic intensity of approximately 65 dB SPL (A), as measured using a sound level meter (Digital Sound level meter 78588, Shinwa Rules Co., Ltd, Japan). Participants were instructed to ignore the sounds played from the speaker and to focus on the fixation point displayed in the centre of the screen. All participants were asked if they recognized any deviation to the sound posterior to all the study.

The background luminance was set to 60 cd/m^2^ to avoid effects of luminance and to extract pupil dilation by auditory deviation. A fixation point was located in the centre of the screen at a visual angle of 0.3 degree.

### Procedure

#### Behavioural auditory distinguishing ability test

A behavioural auditory distinguishing ability test was assessed with a 2AFC (Two-alternative forced choice) task to evaluate subjective /l/-/r/ auditory distinguishing ability (lrADA) in advance of the pupillometry experiment. The word sets used in this test were selected by referring to the experiment by Chang *et al*.^6^. Twenty speech synthesized English words including phoneme /l/ and /r/ each, were generated and controlled in the same way as the experiment stimuli. The number of words from each group was controlled to be equal, and either one in each pair was randomly reproduced by the speaker. The presentation was conducted according to the order of the list shown in Table 1. The participants reported which consonant (/l/ or /r/) was in the presented word by filling out the answer sheet. The answer was then scored by the experimenter. Table 1 shows the word groups including /l/ and /r/ used in this test.

**Table 1:** List of words used in the distinguishing test.

Pair number	Word group containing phoneme /l/		Word group containing phoneme /r/	
	word	IPA	word	IPA
1	**light**	/lάit/	**right**	/rάit/
2	**glass**	/glˈæs/	**grass**	/grˈæs/
3	**lamp**	/lˈæmp/	**ramp**	/rˈæmp/
4	**leach**	/líːtʃ/	**reach**	/ríːtʃ/
5	**flesh**	/fléʃ/	**fresh**	/fréʃ/
6	**pleasant**	/pléznt/	**present**	/préznt/
7	**lane**	/léin/	**rain**	/réin/
8	**lock**	/lάk/	**rock**	/rάk/
9	**fly**	/flάi/	**fry**	/frάi/
10	**let**	/lət/	**ret**	/rɛ́t/
11	**lead**	/léd/	**read**	/ríːd/
12	**leap**	/líːp/	**reap**	/ríːp/
13	**blight**	/blάit/	**bright**	/brάit/
14	**blues**	/blúːz/	**bruise**	/brúːz/
15	**late**	/leit/	**rate**	/ɹeit/
16	**clown**	/klάʊn/	**crown**	/krάʊn/
17	**collect**	/kəlékt/	**correct**	/kərékt/
18	**glow**	/glóʊ/	**grow**	/gróʊ/
19	**lice**	/láis/	**rice**	/rάis/
20	**supplies**	/səpláiz/	**surprise**	/sɚprάiz/

#### Pupillometry task

The task was conducted in a dim lit darkroom and executed in MATLAB 2016a (The MathWorks, Natick, MA, USA) using Psychtoolbox 3^10–12^. Fixation point was displayed continuously at the centre of a liquid-crystal display (LCD) monitor (Display+ +, Cambridge Research Systems Ltd) with resolution of 1920 × 1080 pixels and refresh rate of 120 Hz while auditory stimuli were presented by a speaker (HSTNN-SS01, Hewlett-Packard). Each participant’s chin was fixed at a viewing distance of 920 mm.

**Figure 1:**
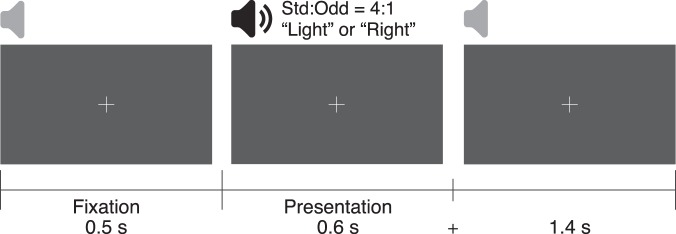
Experimental protocol for one trial. Overview of protocol for a single trial. Fixation point was presented in the centre of the screen through the trial. Participants listened passively to the presented auditory stimuli.

Figure 1 shows the protocol for one trial. In each trial, the fixation point was presented for 500 ms, following which the auditory stimulus was presented for 600 ms. An extra 1,400 ms of fixation was displayed for continuous pupil diameter recording.

Auditory stimuli were randomly presented in one session in order that the presentation ratio of “standard” to “deviant” was 4:1. Participants were instructed to fixate on the fixation point continuously presented on the centre of the screen, while ignoring the auditory stimulus, to put it simply, participants passively listened to the speech stimuli (120 trials per session).

Speech synthesized stimuli pair “light” /lάit/ or “right” /rάit/ by a female speaker were presented as either the standard sound, which is frequently presented, or as the deviant sound, which is the target stimuli in the oddball paradigm. The stimuli and procedure are based on the previous study focusing on auditory English distinction combined with EEG measurement technologies^6^. The two stimuli were presented in pseudo-randomized order, the oddball stimuli presented in low frequency were controlled not to be presented continuously. There were sufficient breaks between each session, and a total of 2 sessions. The standard-deviant condition was counter balanced across participants by the order of sessions (either “light” /lάit/ or “right” /rάit/ were presented as the standard stimuli in the first session). Overall, 240 trials, 192 standard sound trials and 48 deviant sound trials, were conducted. A behavioural auditory distinguishing ability test was performed prior to the experiment to quantitatively evaluate participants’ ability to distinguish English words with /l/ and /r/.

### Pupillometry recording

Pupillary response was recorded binocularly with an Eye tracker (iViewX RED500, SMI SensoMotoric Instruments GmbH Ltd) at a sampling rate of 250 Hz. A nine-point calibration was performed prior to each session. The timing of blinks during pupil diameter recording was not specified to participants, thereby blink interpolation was performed before analysis.

### Analysis of pupil size

The pre-processing and analysis of pupillary response data derived by the eye tracker were conducted with MATLAB 2017b (The MathWorks, Natick, MA, US). The eye blinks were interpolated using Hermite interpolation. Trials with additional artefacts, found by using peak change on the velocity of the pupil response, were excluded from the analysis by thresholding (assuming trials with pupil diameter change more than 0.042 mm/ms as artefacts).

One participant’s pupillary response data was excluded from analyses due to rejection of more than 80% of trials, which could not be interpolated and were thus rejected by thresholding, assuming that data was not acquired properly.

In the time course analysis, the pupil size at stimulus onset in each trial data was normalized relative to the baseline pupil size, following which smoothing of each data point with ±5 sampling points was performed. Baseline pupil size was computed as an average of data collected prior to stimulus onset (stimuli presentation), from −100 ms to 0 ms (presentation onset).

We also calculated the grand-averaged change in pupillary response for each stimuli condition on fixation and stimulus presentation, 2.5 s time course in total. The grand average was computed by the mean of each pupillary response in the two conditions (oddball and standard), and then averaged across participants. So as to confirm whether there is a significantly larger PDR induced by the oddball stimuli, t-tests were used to compare the mean pupil dilation in all time domains between stimuli conditions.

In addition, according to the scores of the behavioural auditory distinguishing ability test performed in advance, the participants were classified into two groups (above-median group and below-median group) and statistical analysis was also performed. Moreover, the correlation coefficient was calculated by simple regression analysis to see if there is a correlation between the pupil response and the yielded lrADA. In this analysis, the mean pupil diameter difference was computed by each individual in the specific time domain related to the PDR, and then the correlation was evaluated.

### Ethical approval and informed consent

All experimental procedures were in accordance with the ethical principles outlined in the Declaration of Helsinki and approved by the Committee for Human Research at Toyohashi University of Technology. Experiment was strictly conducted in accordance with the approved guidelines of the committee. Informed written consent was obtained from participants after procedural details had been explained.

## Results

### Behavioural auditory distinguishing ability test

Twenty-one participants undertook the auditory distinguishing ability test with words shown in Table 1. All participants answered all questions, and the answer sheet was scored by the experimenter. The average score was 13.3 out of 20; standard deviation was 3.65.

The average correct answer rate was 67%. Taking the chance level (50%) into account, these results also suggest the difficulties of distinguishing words including /l/ and /r/ for Japanese. In addition, variation in the distribution of scores (score range: 7–20, SD = 3.65) implies individual differences in subjective English listening ability.

### Pupillometry

**Figure 2:**
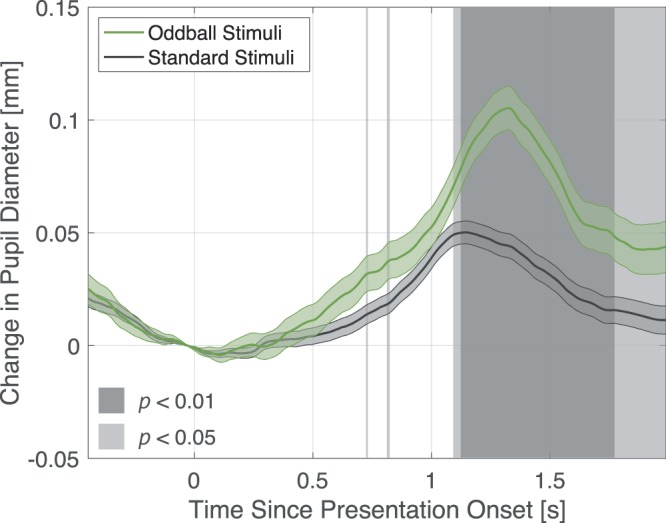
Change in pupil diameter under each stimuli condition. Mean change in pupil diameter from auditory stimulus presentation. Error bars are standard error of the mean. The shaded time domain represents observed significant difference.

Figure 2 shows the grand average of the pupil response between 0.5 s before and 2.0 s after stimulus presentation under each stimulus condition (“standard”, “deviant”).

The shaded area in Fig. 2 represents the period over which a significant difference was observed by the t-test, p < 0.01 in the dark shade on time course approximately over 1.1–1.8 s after the stimulus onset. The pupillary response suggests deviant stimuli (oddball stimuli) induce significant dilation compared to standard stimuli presentation.

Beyond that, the same analysis was conducted against the two classified groups (above-median group and below-median group) according to the lrADA scores of the earlier behavioural distinguishing ability test. Classification was made in order that the number of participants in each group was equal; in particular, the reference point of the division was 14, which is also close to the average score.

**Figure 3:**
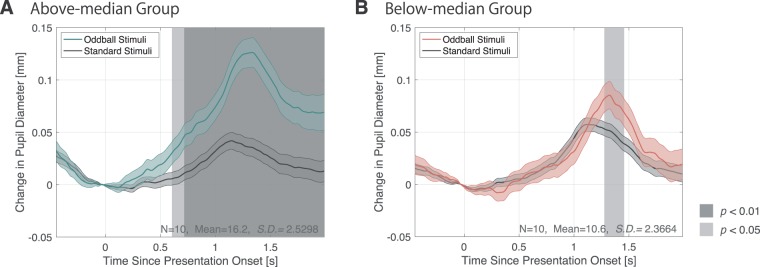
Pupillary response by linguistics distinguish ability. (**A**) Pupil diameter change of the inter-subject average (N = 10) of the high discrimination ability group (above-median group) with high lrADA. Y-axis represents the pupil diameter change from the baseline. Error bars represent the standard error. (**B**) Pupil diameter change of the inter-subject average (N = 10) of the low discrimination ability group (below-median group) according to the behavioural distinguishing ability task. As with (**A**) the error bars are standard error of mean, the shaded area represents the time domain with significant difference by t-test analysis.

Figure 3 shows the grand averaged pupil response by the above-median and below-median groups. As with Fig. 2, the solid error bars represent the standard error of the pupillary response, and the shaded time domain shows where the significant difference was observed.

Specifically, p < 0.01 in the time course was approximately after 0.7 s for the above-median group (Fig. 3A), p < 0.05 was approximately over 1.4–1.5 s for the below-median group. A significant PDR induced by the oddball phoneme stimuli were observed in both groups; moreover, this tendency was particularly evident among the above-median group.

**Figure 4:**
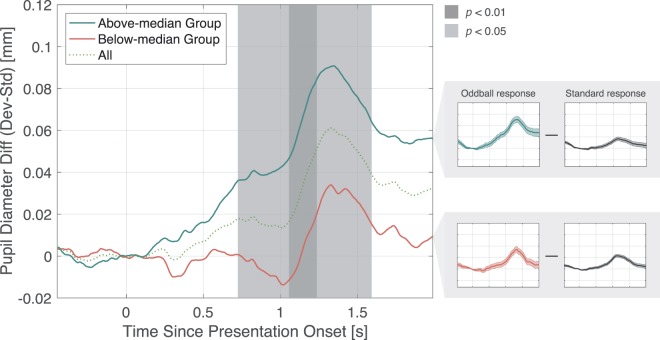
Subtracted pupillary response difference in above-median/below-median group. Calculated mean pupil diameter difference in the oddball and standard stimuli presentation condition. Each solid line represents the subtracted pupil response difference of each classified group. The dotted line is the mean difference for all participants. The shaded time domain represents observed significant difference.

Difference in pupillary responses was obtained by subtracting the pupillary response of the standard stimuli from the deviant stimuli in each group (Fig. 4). The shaded area represents the period over which a significant difference was observed by the t-test. Time course of p < 0.05 is shown approximately over 0.7–1.6 s after the stimulus onset, p < 0.01 approximately over 1.1–1.2. These results suggest the high lrADA group (above-median group) induced a larger pupil diameter difference compared to the below-median group. Furthermore, the pupil diameter difference gradually increases shortly after stimulus onset in the above-median group. PDR, which reflects subjective auditory deviation and unnaturalness is reported to have a peak between 1–2 s of latency^7^; hence, a t-test on the average pupil diameter difference over the 1–2 s time domain in each group was conducted.

**Figure 5:**
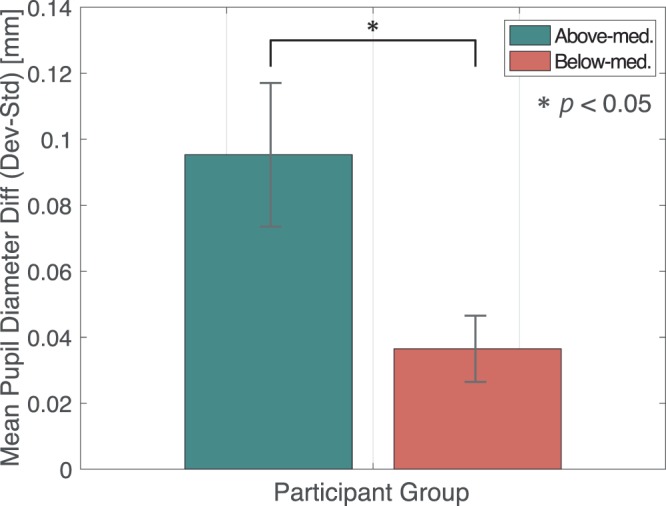
Average pupil diameter difference by groups. Bar graph represents each classified group’s mean pupil change difference between the stimulus conditions in a specific time domain related to the PDR. (1–2 s) The error bars in the figure are standard error.

Figure 5 illustrates the averaged pupil diameter change differences in each group. The error bars in the figure represent the standard error. According to the figure, the average pupillary response difference in each group was *t*(18) = −2.45369, *p* = 0.02455, *Cohen’s d* = 0.53737, and a significant difference was found between the above-median group and the below-median group. (p < 0.05).

### Correlation between pupil diameter and lrADA

The results of pupillary response analysis suggested that the PDR is associated with the participant’s subjective English distinguish ability based on the behavioural test. Therefore, we next assessed the correlation between pupil diameter and subjective English listening ability according to the pre-test score.

**Figure 6:**
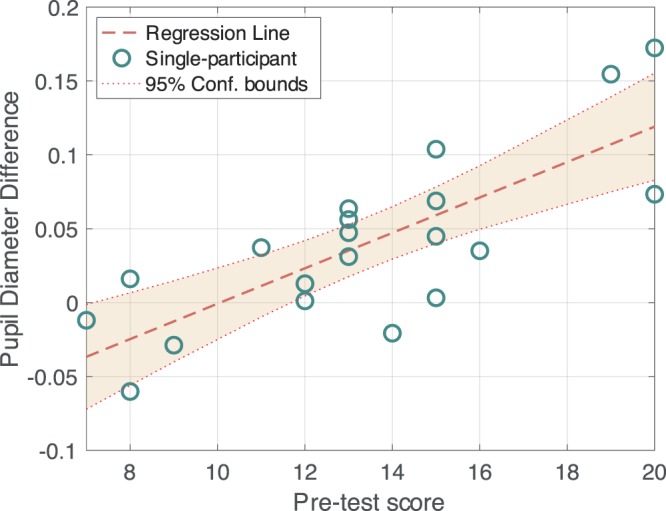
Correlation between pupil diameter and lrADA. Scatter plot represents the correlation between average pupil diameter difference in the oddball/standard stimuli and behavioural test score. Each plot corresponds to one participant. The shaded area represents the 95% confidence interval for the regression, and the dotted line represents each boundary.

Figure 6 presents the simple linear regression analysis. Each plot corresponds to the participant’s average pupil diameter difference in the oddball and standard stimuli presentation condition, over the 1–2 s time domain after the stimulus onset.

By means of single regression analysis based on the least-squares method, a strong correlation between pupil diameter and subjective lrADA (Correlation coefficient R = 0.777 (R^2^ = 0.603), p = 5.63 ×10^–5^) was found. In other words, participants who have higher subjective English distinguish ability tend to have a greater pupil-dilated response when speech sounds including phoneme /l/ and /r/ are presented as deviant stimuli. The coefficient of determination (R^2^) of the regression model suggests the PDR difference induced by the oddball phoneme stimuli can be a predictor of subjective English listening ability. Also, in particular, the confidence interval in Fig. 6 shows the auditory distinguishing ability test score can be estimated approximately within ±2 points with a 95% probability.

## Discussions

The purpose of this research was to establish a method to evaluate participants’ subjective English listening ability including discrimination of /l/ and /r/ by PDR which reflects subjective deviation. A pupillometry experiment was conducted to reveal the correlation of lrADA with PDR.

First, we conducted an English listening discrimination test prior to the experiment to quantitatively evaluate Japanese monolingual participants’ ability to distinguish English words with /l/ and /r/. Then, pupillary response recording during speech sound presentation was performed. As the results showed, a significant PDR was evoked regardless of English speech stimuli; moreover, the PDR was evident among participants with higher lrADA of words including phonemes /l/ and /r/. Additionally, the results suggest PDR difference induced by the oddball phoneme stimuli can be a predictor of Japanese monolingual participants’ subjective English lrADA. Even more, this strong correlation between pupil diameter change and subjective /l/-/r/ distinguishing ability was found in spite of the fact that all individuals were able to easily recognize the difference of the stimuli in the oddball paradigm.

In this experiment, we measured changes in pupil diameter as a subjective deviation to establish a method to quantitatively determine participants’ subjective lrADA of words including phonemes /l/ and /r/. From all classified groups (Figs. 2, 3A, B), a significant dilation was induced by /l/-/r/ speech sounds as deviant stimuli. These dilations could be attributed to PDR on account of dilation latency and pupillary response consistent with the previous research^7–9^. Moreover, results revealed that subjective aspects such as higher English speech sound distinguishing ability, elicits a prominent PDR. Surprisingly, regardless to distinguishing ability, all participants reported the two stimuli were easily distinguishable in the oddball task paradigm. However, as shown in Fig. 5, significant differences were observed in PDR among the groups. Quirins *et al*. has reported these PDR induced by auditory deviation are associated with both passive and active listening tasks in conscious processing. This result also supports the PDR by both groups are induced by the passively presented oddball stimuli^13^.

Previous studies have reported the amplitude of the PDR induced by auditory deviation may be due to the saliency of the sound^7,14^. Although the physical differences in speech stimulation can be easily distinguished in either group, there is a distinction in PDR. Considering these facts, we address the possibility that PDR is sensitive to other higher-order characteristics involved in language processing, such as speech processing related to lrADA.

From the results, the pupil diameter difference gradually increases shortly after stimulus onset only in the above-median group as shown in Fig. 4. In other words, the latency of pupillary response difference induced by the /l/-/r/ auditory stimuli, differed between the lrADA. Since early studies, task-evoked pupillary response has been used as an assay of cognitive effort in perceptual and cognitive tasks^15,16^ and difficult words in a second language have been reported to cause significantly larger dilation compared to simple words^17^. Moreover, from previous research focusing on pupil diameter change as an index of cognitive load affected by language experience, bilingual speakers with higher English proficiency had less cognitive load^18^. The relation between pupillary response and features by words per se have also been reported. Vacchiano *et al*. measured pupil responses to English-word stimuli with different rating values and report that pupil dilation is induced for low-value words^19^. However, as the auditory stimuli were presented passively to the participants, the pupil dilation effect associated with cognitive effort due to bilingualism or less language experience maybe limited to subconscious language processing.

Even more, the cognitive processing and retrieval effort that occur from English word recognition and speech processing in the above-median group is considered to be lower compared to the below-median group. However, the above-median group had a large induced PDR. On the other hand, although the physical difference of the /l/-/r/ stimulus is easily distinguishable in either group, the larger PDR in the above-median group suggests a possibility that individuals with higher English proficiency or distinguishing ability, recognize speech sounds as word stimuli rather than sounds.

Previous research has established that the magnitude of PDR reflects differing levels of cognitive processing. On top of that, higher cognitive factors associated with English proficiency may be contributing to the dynamics difference of pupil response. In fact, Tamási *et al*. has reported that the magnitude of the pupillary dilation is also related with the lexical representation distance by toddlers^20^. Although, these local factors such as English proficiency cannot be extracted by tasks such as the behavioural auditory distinguishing ability test which rely on behavioural responses. Therefore, it might be possible to establish an even more reliable and implicit method to estimate subjective lrADA of English words and English listening abilities from pupil measurement.

However, some limitations should be noted. The word sets used in the behavioural auditory distinguishing ability test were selected by referring to the previous study. Although the stimuli condition in the eye-tracking experiment was counter-balanced across participants to control the dynamics difference of pupillary response which may be influenced by the word frequency effects, the frequency effects in the behavioural auditory distinguishing ability test was not fully considered. Additionally, although the physical properties of the auditory stimuli used in the pupillary study and behavioural auditory distinguishing ability test was carefully controlled, we could not fully deny the possibility that generic auditory sensitivity irrelevant to phonetic categorization may influence the PDR. As speech synthesis stimuli were used in terms of reproducibility, stimuli set in further study should involve multiple utterances by different speakers. Likewise, the feedback in the behavioural auditory distinguishing ability test is limited to a binary (correct/incorrect) assessment of behavioural responses. Additionally, difficulties in auditory distinction of sounds /l/ and /r/ is reported also by Korean monolingual speakers learning English and English speakers learning Mandarin^4,21^. Although, this study has recruited Japanese monolingual participants and did not include any participants with other nationalities or L1 language. To further investigate the relation between participants’ lrADA and general English proficiency, we should take words frequency into account of the behavioural test, and also evaluate the correlation between PDR and English proficiency assessed by other general indicators. Even more, we should further clarify how PDR reflects phonetical aspects in other nationalities, which can lead to a reliable method indicating subjective listening ability on the learning language.

## Conclusions

Our results provide evidence that Japanese monolingual participants’ subjective English lrADA can be estimated by PDR which serves as an index of sound-discrimination ability. In addition, the PDR difference in two groups classified by their listening discrimination ability suggests that PDR might be sensitive to a higher-order characteristic involved in language processing independent of the physical sound aspect.

Two auditory stimuli (“light” /lάit/ and “right” /rάit/) were used in the experiment. Although the results suggest participants’ English lrADA can be estimated just by this stimuli pair, it was difficult to determine whether the specific difference in pupillary response was due to differences in language proficiency or due to differences in the physical sounds aspect. Therefore, future studies should investigate the differences of group dependent characteristics.

Specifically, an additional experiment should include multiple stimuli as shown in Table 1.

By conducting these studies, it will be possible to further elucidate the phenomenon discovered here and to investigate the cognitive mechanism involved in language processing.

## Data Availability

All dataset generated during this study are included in this article and are or analysed data are fully available from the corresponding author on reasonable request.
